# Fabrication of TiO_2_ Nanotube Arrays by Progressive Anodization of Ti Thin Film on Insulated Substrates

**DOI:** 10.3390/ma18061219

**Published:** 2025-03-09

**Authors:** Chao-Ching Chiang, Jian-Sian Li, Hsiao-Hsuan Wan, Fan Ren, Josephine F. Esquivel-Upshaw

**Affiliations:** 1 Department of Chemical Engineering, College of Engineering, University of Florida, Gainesville, FL 32611, USA; cchiang@ufl.edu (C.-C.C.); jiansianli@ufl.edu (J.-S.L.); hwan@ufl.edu (H.-H.W.); fren@che.ufl.edu (F.R.); 2 Department of Restorative Dental Sciences, Division of Prosthodontics, College of Dentistry, University of Florida, Gainesville, FL 32610, USA

**Keywords:** nanotube array, anodization, thin film

## Abstract

Titanium (Ti) thin films deposited on insulated substrates were progressively anodized and formed titanium dioxide (TiO_2_) nanotube arrays on the surface through a customized anodization tool designed to improve the uniformity and diameters of the nanotubes. With a motorized vertical moving arm attached to the anode, the sample was gradually submerged into the electrolyte at a controlled speed alongside the continuous anodization from the edge to the center to prevent the discontinuation of the conductive Ti layer and its nanotube surface. The effects of Ti deposition rate, anodization voltage, NH_4_F concentration, and post-etching conditions on nanotube morphology were also explored. Scanning electron microscopy (SEM) analysis revealed that smaller Ti grain sizes, higher anodization voltages, higher electrolyte concentrations, and optimized post-etching times produce uniform, mature nanotubes with larger diameters, which are crucial for practical applications. This work enhances the applicability of nanotube surfaces with non-conductive substrates, such as Zirconia dental implants, and establishes a foundation for future process optimizations.

## 1. Introduction

The exploration of anodization of valve metals, particularly titanium, has emerged as a fascinating area of research with versatile applications in various scientific domains. These anodized materials, characterized by highly uniform and regular porous structures, serve as templates for creating materials with well-defined geometries. TiO_2_ nanotubes, produced through anodization, have garnered significant importance due to their diverse applications. TiO_2_ nanotubes possess desirable properties such as chemical and thermal stability, catalytic activity, high refractive index, and biocompatibility, making them suitable for applications in electronics, electrochromic devices, gas sensors, biomedical devices, solar cells, and photocatalysis [1,2,3,4,5,6,7,8,9,10,11].

The generation of TiO_2_ nanotubes and the preference for anodization as the method of choice offer an intriguing exploration into controlled nanostructuring. Anodization stands out as a versatile and reliable approach for growing TiO_2_ nanotubes, providing distinct advantages in terms of precision and tunability [12,13,14]. Through this process, titanium can be anodized either in bulk or as a thin film, with each approach offering unique possibilities. Anodizing titanium in bulk allows for the detachment of the titania layer from the residual titanium metal, serving as a versatile template for diverse applications [15,16,17]. The flexibility of anodization parameters, such as voltage, temperature, and solution composition, facilitates the creation of a wide range of tunable morphologies within the resulting nanoporous titania, notably including nanotubes [18,19,20,21,22,23,24]. The capability to control crucial aspects like tube diameter is a notable advantage, with achievable pore diameters ranging from approximately 10 nm to 350 nm [25,26,27,28,29]. Moreover, this method provides the opportunity to fabricate various exotic titanium oxide structures by adjusting the anodization conditions, including nanopillars, nanowires, multilayered structures, and bamboo nanotubes [30,31,32,33,34,35,36]. The inherent versatility and controllability of anodization make it a preferred method for the precise growth of TiO_2_ nanotubes with diverse morphologies.

One of the most promising applications of TiO_2_ nanotube surfaces lies in their use for antibacterial dental implant coatings, providing a textured surface that enhances antibacterial properties [37,38,39,40,41,42]. While titanium remains the most common material for dental implants, the rise of non-metallic materials like zirconium dioxide (Zirconia) offers distinct advantages, particularly for patients with metal sensitivities. Zirconia’s non-metallic composition ensures biocompatibility, reducing the risk of allergic reactions [43,44,45,46]. Additionally, zirconia implants provide superior esthetic benefits, closely resembling the natural appearance of teeth and blending seamlessly with the surrounding tissue. This makes them particularly suitable for patients with thin gingival biotypes, as they are less likely to be visible through the gum line [47,48,49]. The innovative combination of TiO_2_ nanotube coating on Zirconia dental implants holds promise for an all-encompassing material choice for dental implants.

Clinical and animal studies have also shown that zirconia implants exhibit comparable or even superior osseointegration and antibacterial properties compared to titanium implants. These features contribute to better prevention of peri-implantitis, enhanced bone healing, and increased long-term implant stability. Depprich et al. and Gahlert et al. demonstrated in vivo that zirconia implants with modified surfaces achieve osseointegration on par with titanium implants [50,51]. Sollazzo et al. further showed that titanium implants coated with zirconia resulted in a significantly higher bone–implant contact percentage compared to pure titanium implants [52]. Clever et al. found that soft tissues around titanium implants exhibited a stronger inflammatory response to experimental plaque accumulation, with elevated levels of IL-1β, IL-6, and TNF-α, compared to zirconia implants, highlighting zirconia’s superior antibacterial properties [53].

The uniformity and the diameter of the nanotubes on the surface of dental implants significantly impact their antibacterial properties [41,42,54,55]. The typical TiO_2_ anodization process starts from fully submerging the Ti thin film into a fluorine-based electrolyte. When an anodization voltage is applied to the thin film on an insulated substrate, a fully anodized region forms at the surface of the electrolyte since the higher current density closer to the surface completely anodizes the Ti thin film, blocking the current and preventing further anodization of the rest of the sample. This results in a discontinuation of nanotubes on the sample surface with low uniformity and limited diameters. Tang et al., Kılınç et al., and Mor et al. [26,56,57,58] demonstrated the anodization of Ti thin film deposited on glass substrates but only achieved nanotube diameters of less than 100 nm. Therefore, developing a specialized method to anodize Ti thin film deposited on non-conductive substrates is crucial.

The model elucidating the theory behind the formation of anodized TiO_2_ nanotube arrays has been well known, positing that Ti anodization results from the interplay between electrochemical oxide formation and chemical dissolution of the oxide by fluoride ions [59,60,61,62,63]. Anodization requires placing the Ti material in a conductive analyte alongside a counter electrode. In the absence of fluoride ions (F^−^), a thin barrier metal oxide is first produced on the metal surface through the reaction:

Ti + 2H_2_O -> TiO_2_ + 4H^+^ + 4e^−^ (1)

This reaction can be augmented by applying an electric field, facilitating ion transport (O^2−^ and Ti^4+^ ions) through the growing oxide. However, as the oxide layer thickens during anodization, the electric field across the film diminishes, constraining the oxidation process and causing a decrease in the oxidation current. At the oxide/electrolyte interface, Ti^4+^ ions are not rendered soluble by complexation, leading to the precipitation of a loose and porous hydroxide layer (Ti(OH)_x_O_y_) that hinders further diffusion. In the presence of fluoride ions, two effects alter the scenario.

(i) Direct complexation with transported cations at the oxide electrolyte interface, preventing Ti(OH)_x_O_y_ precipitation:

Ti^4+^ + 6F^−^ → [TiF_6_]^2−^ (2)

(ii) Reaction with the oxide to form water-soluble [TiF_6_]^2−^ complexes, leading to dissolution and breakdown of the barrier layer:

TiO_2_ + 6F^−^ + 4H^+^ → [TiF_6_]^2−^ + 2H_2_O (3)

This leads to continuous etching, resulting in an initial increase in current. Over time, the rate of titanium oxide growth, assisted by the electric field, equals the rate of dissolution by fluoride ions, leading to a constant barrier layer thickness. The current eventually decreases due to factors such as a reduction in the diffusion of fluoride-containing species into and out of the tubes, or when the conductivity of the whole thin film is no longer high enough to support the current itself.

This study introduces a novel progressive anodization technique designed to fabricate TiO_2_ nanotube arrays on non-conductive substrates, such as glass and zirconia, which has been a longstanding challenge in the field. While previous research has successfully demonstrated anodization on conductive substrates such as titanium and ITO-coated glass, achieving uniform nanotube growth on insulating materials has remained problematic due to the discontinuity in the conductive Ti layer. The key innovation in this work is the implementation of a motorized vertical moving anode that enables controlled submersion of the sample into the electrolyte, ensuring uniform anodization and preventing early termination of the reaction. This progressive anodization approach eliminates the common issue of inhomogeneous nanotube formation observed in past studies and allows for precise control over nanotube diameter and uniformity. Furthermore, this study provides a comprehensive analysis of the effects of Ti deposition rate, anodization voltage, NH_4_F concentration, and post-etching conditions, which are critical parameters for optimizing nanotube morphology and have not been systematically studied together in previous works.

## 2. Materials and Methods

**Figure 1:**
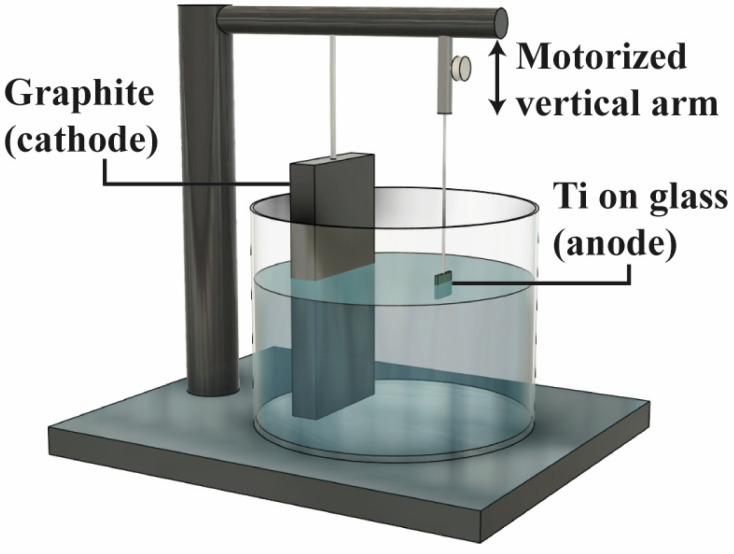
Illustration of the customized anodization tool configuration.

The titanium thin film employed in this study was prepared through electron beam evaporation. Using a high-purity titanium target, a 300 µm layer of titanium was deposited onto glass substrates, employing two different beam powers and the resulting deposition rates of 0.5 Å/s and 3 Å/s. These rates were selected to investigate their impact on grain size and the resulting nanotube morphology. Experiments were conducted on microscope glass slides (Thermo Fisher Scientific, Waltham, MA, USA) due to their insulating properties, which simulate the characteristics of zirconia dental implants. Standard cleaning protocols with acetone (Thermo Fisher Scientific, Waltham, MA, USA), IPA (Thermo Fisher Scientific, Waltham, MA, USA), and nitrogen (Airgas, Radnor, PA, USA) ensured surface consistency. Anodization was conducted in a 95 wt% ethylene glycol (Sigma-Aldrich, St. Louis, MO, USA) solution containing varying concentrations (0.5%, 1%, 2%, and 3%) of ammonium fluoride (NH_4_F) (Sigma-Aldrich, St. Louis, MO, USA), with distilled water constituting the remaining composition. Given the non-conductive nature of the glass substrate, a specially designed anodization device, illustrated in Figure 1, was utilized to ensure a consistent and uniform anodization process across the entire sample surface. The device featured a cylindrical electrochemical reactor chamber incorporating a graphite cathode and a vertically motorized anode connected to the as-deposited Ti-on-glass sample. Both electrodes were linked to a 30 to 90 V DC power supply (Keysight, Santa Rosa, CA, USA). The mobile anode was connected to a 3D printed connection shaft affixed to a high torque, low rpm motor powered by an additional power supply, where the vertical moving speed of the anode sample can be precisely controlled. This mechanism regulated the speed at which the sample was gradually immersed into the electrolyte.

## 3. Results and Discussion

**Figure 2:**
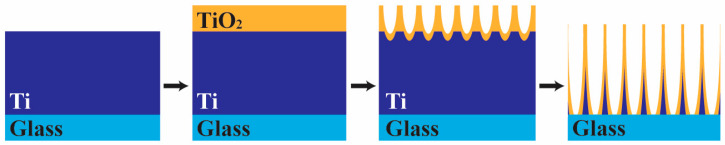
Schematic of the anodization process of Ti thin film deposited on a glass substrate.

The schematic diagram of the anodization process and mechanism in this work is depicted in Figure 2. In the early stages of anodization, Equation (1) results in the formation of a thin oxide layer on the titanium sheet, causing a rapid reduction in current density due to its poor electrical conductivity. Under sufficient applied voltage, electric field-assisted reduction occurs at the TiO_2_/Ti interface with Equations (2) and (3), resulting in the etching of the TiO_2_. Oxygen ions (O^2−^) transport from the solution to the oxide layer, while titanium ions (Ti^4+^) move from the titanium to the oxide/solution interface, dissolving into the solution. This process leads to a continuous increase in the depth of the porous structure, causing the formation of an ordered nanotube array vertically oriented to the substrate. Chemical dissolution of the TiO_2_ with Equation (3) simultaneously reduces the thickness of the nanotube wall, increasing the diameter of the nanotubes. The length of TiO_2_ nanotube arrays continues to increase until the bottom of the tubes touches the non-conductive glass substrate, where the discontinuation of the conductive Ti layer ceases the current-driven reactions.

**Figure 3:**
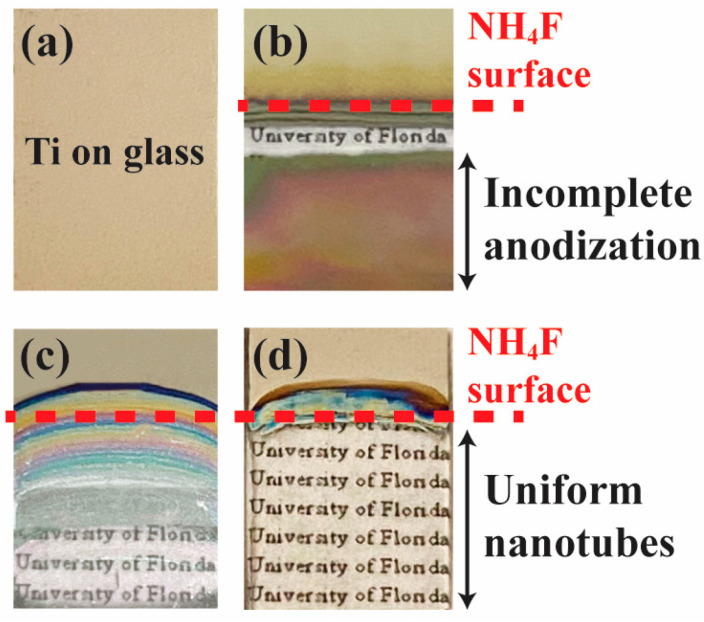
Photographs of Ti thin film on a glass substrate under various anodization conditions. (**a**) The sample before anodization. (**b**) Fixed anode where disrupted Ti continuity leads to incomplete nanotube formation. (**c**) Progressive anodization with excessive anode speed results in shallow nanotubes near the interface. (**d**) Optimized progressive anodization where the anode speed matches the anodization rate, producing a uniform nanotube array with full anodization.

The effect of utilizing the customized anodization tool with controllable anode submerging speed on sample surface uniformity is shown in Figure 3. A sample consisting of Ti thin film deposited on a glass substrate, as depicted in Figure 3a, underwent the traditional anodization process with a fixed anode, fully submerged in the NH_4_F electrolyte before applying voltage. The resulting appearance in Figure 3b shows a band of transparent nanotube region formed at the surface of the electrolyte due to the higher current density closer to the wire connection. This disrupted the continuity of the conductive Ti layer, preventing further anodization and leaving the rest of the sample with incomplete nanotube growth. To address this issue, the anode placement was changed from a fixed position to one that moves vertically at a controllable speed, allowing anodization to progress from the edge of the sample upward as local nanotubes grow to their full length. A sample anodized with this new mechanism using the customized tool is shown in Figure 3c. The dashed line indicates the final surface level of the electrolyte at the end of the anodization process, where the speed of the moving arm exceeded the anodization rate. This mismatch resulted in shallower nanotube depths near the air–electrolyte interface due to insufficient submerging time. Finally, Figure 3d demonstrates the optimized result, where the speed of the sample submerging aligned precisely with the anodization rate. This produced a uniform nanotube array surface morphology, as evidenced by the transparent and consistent appearance across the entire region under the electrolyte. The success of the fully anodized nanotube surface with excellent uniformity highlights the effectiveness of the improved progressive anodization mechanism.

**Figure 4:**
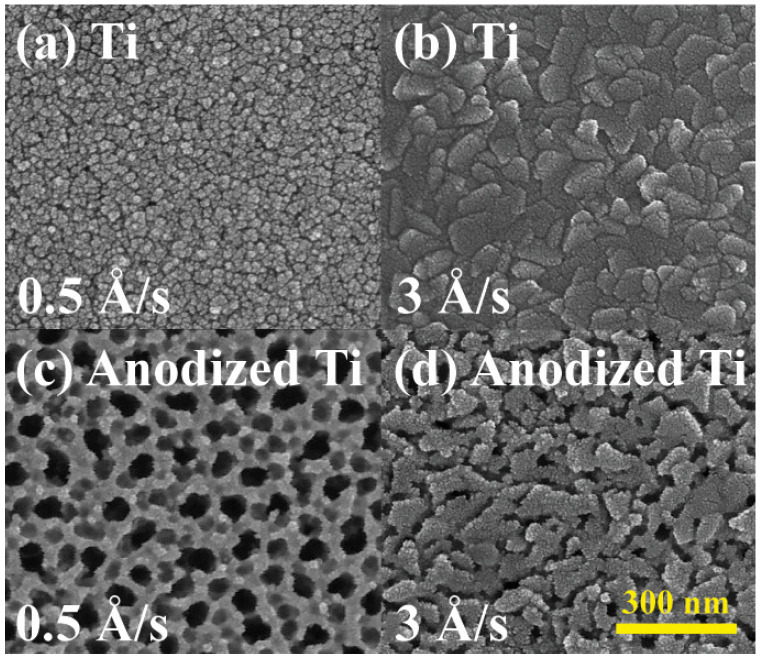
Surface morphology of Ti thin film with the deposition rate of (**a**) 0.5 Å/s and (**b**) 3 Å/s and the nanotube formation after anodization process on Ti thin film with the deposition rate of (**c**) 0.5 Å/s and (**d**) 3 Å/s.

The correlation between the deposition rate in electron beam evaporation and the resulting grain size and surface roughness of the metal thin film has been robustly established [64,65,66,67,68,69]. Lower deposition rates produced smaller, uniform grains, conducive to dense nanotube formation, while higher rates resulted in larger grains, limiting uniformity. Figure 4 illustrates the comparison between titanium thin films deposited via electron beam at two distinct rates, namely 0.5 Å/s and 3 Å/s, and their corresponding anodized nanotube surface morphologies in SEM images. All the other anodization conditions, such as sample size, anode moving speed, NH_4_F concentration, and applied voltage, remained constant. At the slower deposition rate of 0.5 Å/s, the grain size was approximately 10 nm in diameter and exhibited an even dispersion in size. Conversely, the higher deposition rate of 3 Å/s resulted in an increased grain size of the titanium thin film to around 100 nm on average, accompanied by greater variation in grain sizes. Along the grain boundaries, the sharper morphology of the metal surface induces faster rates for the anodization reactions due to the larger specific surface area, defining the starting locations for the growth of the nanotubes. The thin film with the higher deposition rate, composed of larger chunks, experienced limitations in the formation of well-aligned nanotubes with larger diameters since the total grain boundary length per area is short. In contrast, the titanium thin film with a smaller deposition rate and corresponding grain size demonstrated denser and longer grain boundaries, resulting in more concentrated and uniform nanotube arrays after the anodization. In summary, the grain size significantly influenced nanotube uniformity. Films deposited at 0.5 Å/s yielded finer grains with increased grain boundary density, promoting uniform nanotube arrays. Conversely, films deposited at 3 Å/s had coarser grains, resulting in less uniform nanotube distribution due to reduced nucleation sites.

**Figure 5:**
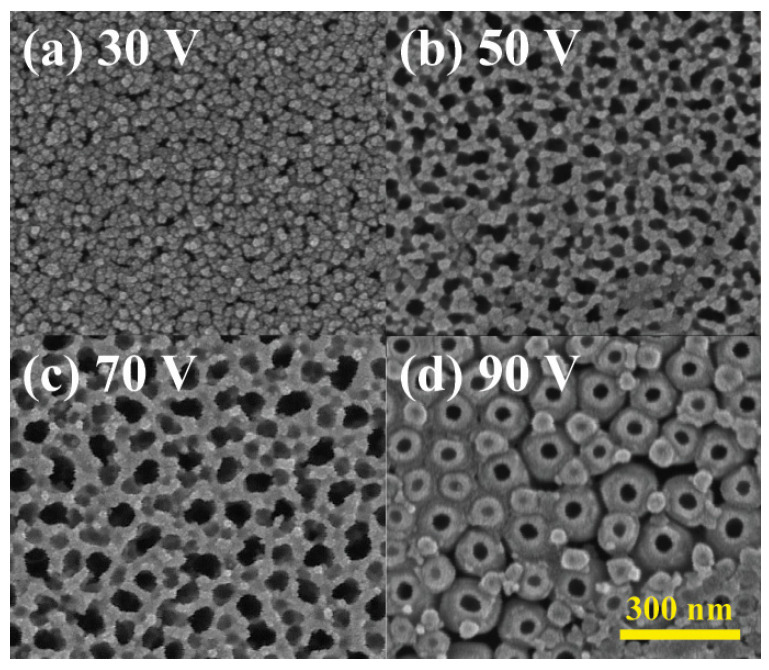
Surface morphology of anodized TiO_2_ nanotubes with NH_4_F concentration of 0.5% and voltage of (**a**) 30 V, (**b**) 50 V, (**c**) 70 V, and (**d**) 90 V.

To explore the impact of anodization voltage on the diameter and surface characteristics of the nanotube array, four distinct voltages—30, 50, 70, and 90 volts—were employed for anodizing titanium thin films on glass substrates. The titanium deposition rate was maintained at 0.5 Å/s, a rate previously determined to be optimal. Anode movement speed and NH_4_F electrolyte concentration were held constant at 0.5 mm/min and 1 wt%. The resulting SEM images in Figure 5 depict a positive correlation between nanotube density/size and anodization voltage due to the enhancement of both the oxidation and etching rate. Additionally, higher voltages led to increased circularity, indicating more matured anodization reactions with elevated current densities. Investigation of higher voltages than 90 V was also performed but the heat and bubble generated during the anodization peeled off the nanotube layers from the substrate.

**Figure 6:**
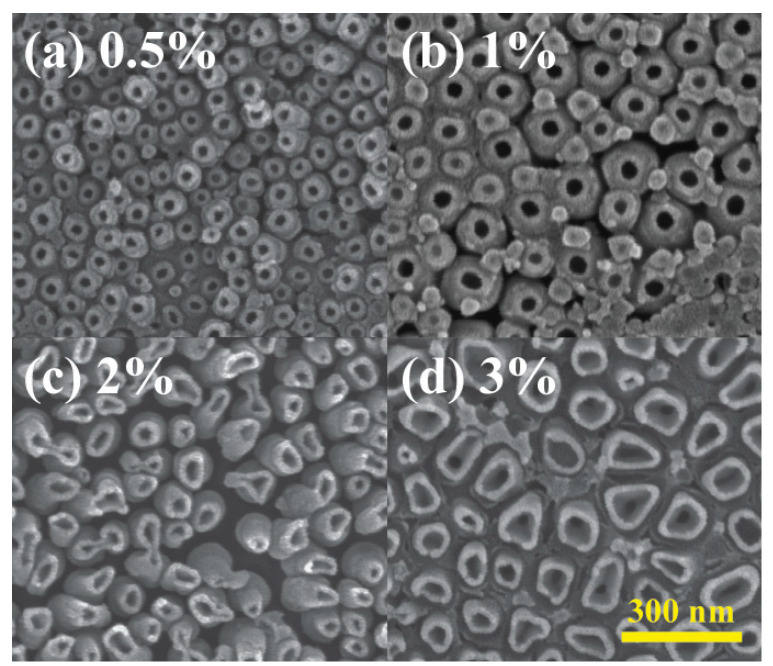
Surface morphology of anodized TiO_2_ nanotubes with NH_4_F concentration of (**a**) 0.5%, (**b**) 1%, (**c**) 2%, and (**d**) 3% and voltage of 90 V.

**Figure 7:**
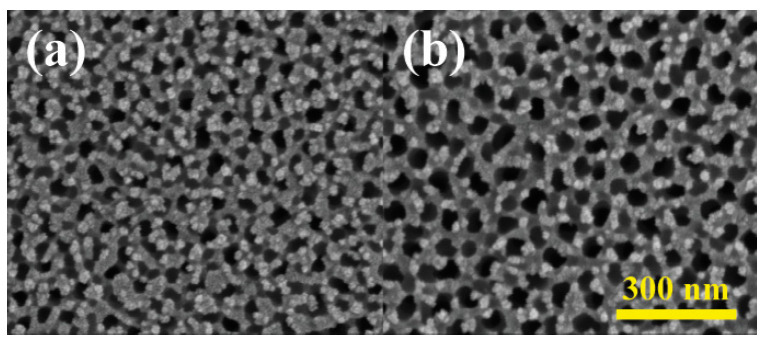
Surface morphology of anodized TiO_2_ nanotubes (**a**) before and (**b**) after soaking in 3% NH_4_F for 30 min.

The influence of NH_4_F electrolyte concentrations on nanotube array surface morphology was also explored in Figure 6. Four concentrations, 0.5, 1, 2, and 3 wt% were investigated, each yielding nanotube inner diameters of 50, 80, 100, and 120 nm. Similarly to the effect of different voltages, nanotube size exhibited a positive correlation with NH_4_F concentrations, attributed to accelerated etching and dissolution of the TiO_2_ barrier layer. Higher electrolyte concentrations also lead to thinner side walls of the nanotubes since the etching part of the reactions dominated more. The same phenomenon could also be observed when the sample with anodized TiO_2_ nanotubes was soaked in 3% NH_4_F electrolyte again for 30 min without applying voltage. As shown in Figure 7, the size of the nanotubes increased 100% due to the continuing etching of the reactions coupled with the absence of electric-field-driven oxidation.

The comprehensive overview of the experimental findings and comparison with literature numbers are presented as tables. Table 1 organizes the detailed results of nanotube formation under various process conditions, such as deposition rates, anodization voltages, NH_4_F concentrations, and post-soaking treatments. This highlights the significant influence of each parameter on the size and uniformity of the nanotubes, demonstrating the effectiveness of the optimized progressive anodization method. The formation of TiO_2_ nanotubes during anodization is governed by the delicate balance between electrochemical oxide formation and chemical dissolution. This simultaneous growth and dissolution creates a porous structure. The balance between these reactions ensures uniform nanotube growth. Deviations, such as excessive dissolution or insufficient oxide formation, disrupt the process and lead to irregular morphologies. These mechanisms are critical for optimizing the anodization process, especially for achieving uniform nanotube arrays on non-conductive substrates like glass or zirconia. Table 2 further underscores the novelty of this study by contrasting this study with those of previous works. Notably, the optimized approach in this study achieves larger nanotube sizes with superior uniformity on non-conductive substrates compared to earlier methods. This advancement addresses the longstanding challenge of achieving high-quality nanotubes on insulating substrates, which expands their applicability to diverse fields such as biomedical devices and sensors.

## 4. Conclusions

The fabrication of TiO_2_ nanotubes through the anodization of Ti thin films on insulated substrates offers a versatile and controlled nanostructuring approach. The anodization process, governed by the interplay of electrochemical oxide formation and chemical dissolution, enables the precise growth of TiO_2_ nanotubes with diverse morphologies. By employing a specially designed anodization tool, the study ensured consistency and uniformity in the anodization process of thin films deposited on non-conductive glass substrates. This work revealed that slower Ti deposition rates, higher anodization voltages, and higher NH_4_F concentrations, combined with post-etching treatments, result in more uniform and mature nanotubes with larger diameters. Compared to previous methods with conductive substrates, the optimized approach demonstrated the ability to fabricate larger and more uniform nanotubes on non-conductive substrates, addressing a key challenge in the field. The findings of this study open new possibilities for the application of TiO_2_ nanotube arrays on non-conductive substrates, particularly in biomedical and electronic fields. One of the most promising applications is the enhancement of zirconia-based dental implants. By integrating TiO_2_ nanotubes onto zirconia surfaces, it is possible to improve implant osseointegration and antibacterial properties, reducing the risk of peri-implantitis and enhancing long-term implant stability. Additionally, the uniform nanotube arrays achieved through progressive anodization could be utilized in biosensors for real-time diagnostics, where enhanced surface area and charge transport properties are crucial for improved sensitivity and performance. Beyond biomedical applications, the methodology developed in this research can be extended to energy-related technologies. TiO_2_ nanotube arrays are widely recognized for their photocatalytic properties, and their fabrication on non-conductive substrates could lead to advancements in self-cleaning coatings, water purification systems, and high-efficiency dye-sensitized solar cells. The ability to precisely control nanotube dimensions through the optimized anodization process also presents opportunities for developing next-generation nanostructured electrodes in energy storage devices, such as lithium-ion batteries and supercapacitors. Future research will focus on expanding the applicability of this technique to other insulating materials, optimizing nanotube surface functionalization for specific applications, and integrating real-time monitoring mechanisms to further refine the anodization process. Additionally, investigations into the long-term stability and mechanical properties of nanotube-coated zirconia implants will be conducted to validate their suitability for clinical applications.

## Figures and Tables

**Table 1:** Summary of nanotube sizes under various experimental conditions, including deposition rates, anodization voltages, NH_4_F concentrations, and post-soaking treatments.

Condition	Process Parameters	Nanotube Size (nm)
Ti film deposition rates	0.5 Å/s	10
	3 Å/s	100
Anodization voltages	30 V	50
	50 V	80
	70 V	100
	90 V	120
NH_4_F concentrations	0.5%	50
	1%	80
	2%	100
	3%	120
Post-soaking in 3% NH_4_F	Pre-soaking	50
	Post-soaking	100

**Table 2:** Comparison of nanotube sizes and conditions from this study with previous works, highlighting the novelty and advantages of the optimized progressive anodization technique.

Study	Substrate	Nanotube Size (nm)
This study	Glass (non-conductive)	50–120 (optimized conditions)
[26]	ITO glass (conductive)	<100
[57]	Ti thin film	~60–90
[58]	Ti thin film	~30–50

## Data Availability

The original contributions presented in this study are included in the article. Further inquiries can be directed to the corresponding author.
